# Ladies of the past Generation and the Present

**Published:** 1869-11

**Authors:** 


### LADIES OF THE PAST GENERATION AND THE PRESENT.

The *Times*, in a biographical sketch of the late Lady Palmerston, says, "Her childhood and girlhood must be supposed to have passed like those of other young ladies of her rank, and her education, except what must have accrued imperceptibly from maternal influences, to be the same. Female education did not then aim at crowding the memory with what is called useful knowledge; its chief objects were grace and accomplishments, and the results were seen in individuality and variety of character, in the freer development of the natural faculties, in greater ease, freshness and elasticity. Women of quality differed like their handwriting, which is now uniform and generic instead of personal and peculiar. Such, at least, is the broad inference we should draw from the many bright illustrations that have survived to our day, beginning with the one who has given occasion for these remarks."

